# On the Origin of Alkali-Catalyzed Aromatization of Phenols

**DOI:** 10.3390/polym11071119

**Published:** 2019-07-02

**Authors:** Yu Ji, Qiang Yao, Yueying Zhao, Weihong Cao

**Affiliations:** 1 Ningbo Institute of Materials Technology and Engineering, Chinese Academy of Sciences, Ningbo 315201, China; 2 University of Chinese Academy of Sciences, Beijing 100049, China

**Keywords:** phenolate, thermal degradation, radical anion, aromaticity, aromatization

## Abstract

To gain an insight of the chemistry in the alkali-promoted aromatization of oxygen-containing heavily aromatic polymers or biomass; thermal degradations of sodium phenolates with different substituents have been investigated. The -ONa group strongly destabilizes the phenolates. The thermal stability of phenolates is largely in parallel with bond strengths of Ar substituents. De-substituents and the removal of aromatic hydrogens are dominant reactions in the main degradation step. CO is formed only at a very late stage. This degradation pattern is completely different from that of phenol. To account for this distinctive decomposition; a mechanism involving an unprecedented formation of an aromatic carbon radical anion generated from the homolytic cleavage of Ar substituent (or Ar–H) in keto forms has been proposed. The homolytic cleavage of Ar substituent (or Ar–H) is facilitated by the strong electron-donating ability of the oxygen anion. A set of free-radical reactions involved in the alkali-catalyzed aromatization have been established.

## 1. Introduction

Aromatization is a fundamental process in the carbonization of oxygen-containing char-forming polymers and biomass [1,2]. Numerous studies have demonstrated that the aromatization is significantly affected by the presence of alkali catalysts [3,4,5,6]. For example, at an astoundingly low loading level of 0.1%, potassium 3-(phenylsulfonyl)benzenesulfonate (KSS) enables bisphenol A polycarbonate (PC) to achieve a remarkable UL-94 V0 rating by accelerating the latter’s aromatization process [3]. Although the scale of charring acceleration is quite striking, the generally observed different degradation products and/or their distribution in the presence of alkalis suggest that alkali ions are more than to merely enhance the reaction rate [7,8].

To understand the action of alkalis, considerable efforts have been devoted to investigating the thermal degradation of polymers or biomass in the presence or absence of alkalis. Significant progress has been made in the clarification of the degradation products or intermediate structures, and the action of alkali ions on the primary reactions such as dehydration, esterification, and decarboxylation in early degradation stages have been well documented [9,10,11,12,13]. However, the use of complex raw materials might confound the alkalis’ roles in the later aromatization process. So far, the origin of catalytic action of alkali ions on the aromatization largely remains speculative. 

One way to potentially elucidate the aromatization process promoted by alkalis is through studying model compounds which can represent the char-forming precursors. In this regard, phenols are highly valuable candidates since they have been established as one of key intermediates in charring of oxygenated heavily aromatic polymers such as PC and lignin [14,15,16]. However, although the thermal degradation of phenols has been extensively investigated, little has been known about that of alkali phenolates. We surmised that a comparison of their degradation pathways might shed lights on the origin of the alkali-promoted aromatization of phenols and subsequently polymers. 

In this work we have studied the thermal degradation of a series of sodium phenolates with different substituents. We have found that the thermal degradation of phenolates is fundamentally different from that of phenols. The -ONa group remarkably accelerates the removal of aromatic hydrogens and de-substituents of phenolates. Based on the degradation pattern of phenolates, a new mechanism involving a formation of an aromatic carbon radical anion as a key step has been proposed. A set of free-radical chemistry has been established for the aromatization of phenolates. The origin of alkalis’ action on the aromatization of oxygen-containing polymers has been clarified. 

## 2. Experimental

### 2.1. Materials

Sodium phenolate was bought from Energy Chemical Co. Ltd. (Shanghai, China). *p*-Phenoxyphenol and sodium salicylate were purchased from Aladdin Co. (Shanghai, China). Sodium hydroxide, sodium benzoate, *m*-cresol, and *p*-cresol were obtained from Sinopharm Chemical Reagent Co. Ltd. (Shanghai, China). All materials were used without further purification. 

### 2.2. Preparations of Sodium Phenolates

Sodium salts of *p*-phenoxyphenol and *p*- or *m*-cresol were prepared by neutralization with aqueous sodium hydroxide followed by drying in a vacuum oven to a constant weight.

### 2.3. Characterizations

Thermal gravimetric analysis (TGA) experiments of sodium phenolates were conducted on a Q500 thermogravimetric instrument. About 5 mg samples were loaded in a platinum pan and then heated from 50 to 800 °C at a rate of 10 °C/min in nitrogen (60 mL/min).

Fourier transform infrared spectra (FTIR) of the residues collected in the TGA runs after the main degradation step were recorded on a Cary 660 FTIR spectrometer (Agilent Technologies, Santa clara, CA, USA) interfaced to a GladiATR (Pike Technologies, Fitchburg, WI, USA) with diamond crystal at 4 cm^−1^ resolution.

Thermogravimetric analysis/infrared spectra (TGA-IR) of sodium salts were performed on either a TGA/DSC1 analyzer (Mettler-Toledo, Switzerland) interfaced to a Nicolet 6700 FTIR spectrophotometer (Thermo-fisher, Waltham, MA, USA) (for sodium phenolate, sodium *p*-cresolate and sodium *m*-cresolate) or a TGA 8000 analyzer (PerkinElmer, Columbus, OH, USA) interfaced to a Spectrum Two FTIR spectrophotometer (PerkinElmer, Columbus, OH, USA) (for sodium *p*-phenoxyphenolate, sodium salicylate). The temperatures of the transferring line between TGA and FTIR were set to 200 and 270 °C, respectively. Samples of 3–5 mg were heated from 50 to 800 °C at a heating rate of 10 °C/min under a nitrogen atmosphere with a flow rate of 50 mL/min. The spectra were recorded at 4 cm^−1^ resolution every 40 s for 85 min.

Thermogravimetric analysis/mass spectrum (TGA-MS) of sodium phenolate was conducted on a TGA 8000 analyzer interfaced to a Clarus SQ 8 T mass spectrometer (PerkinElmer, Waltham, MA, USA). The temperature of the transferring line between TGA and MS was set at 270 °C. About 3 mg sample was heated from 50 to 800 °C at a heating rate of 10 °C /min in nitrogen (60 mL/min).

Gas chromatograph of collected off-gas of sodium phenolate in the TGA experiment was performed on a 7890B gas chromatograph (Agilent Technologies, Santa clara, CA, USA). The temperatures of column box, thermal conductivity detector (TCD) and valve box were independently 60, 240 and 60 °C. The flow rates of helium in TCD and pneumatics control module (PCM) C were set to 40 and 28 mL/min, respectively. The pressure of PCM C was 18 psi. Valve 1 opens at 0.01 min and closes at 3.3 min, and Valve 2 opens at 1.6 min and closes at 9 min.

## 3. Results and Discussion

### 3.1. TGA of Sodium Phenolates

**Figure 1:**
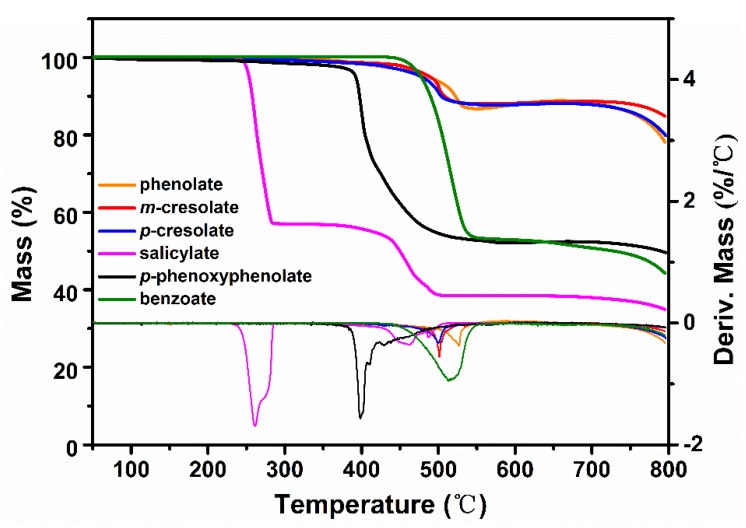
The thermogravemtric analyses of sodium phenolates under N_2_.

The thermogravimetric analyses of various sodium phenolates and sodium benzoate are shown in Figure 1. T_1%_, T_5%_, and T_max_, which are the temperatures at 1%, 5% and maximum mass losses respectively, and mass losses of the main degradation steps are listed in Table 1.

From the shape of TGA curves and the values of T_1%_, T_5%_, and T_max_, it is clear that sodium salicylate is the least thermally stable, followed consecutively by *p*-phenoxyphenolate, *p*-cresolate, and *m*-cresolate with sodium phenolate exhibiting the highest thermal stability. This order of the thermal stability is generally in parallel with the bond strength of NaOPh-substituent (Ar substituent). For example, the bond dissociation energy (BDE) of Ph–H (464.0 kJ/mol) is larger than that of Ph–CH_3_ (425.9 kJ/mol) [17]. While the exact value of BDE of NaOPh–OPh is not available, the radical stability of PhO· is higher than that of CH_3_· so it is conceivable that the bond strength of Ar–OPh is weaker than Ar-CH_3_. On the other hand, although Ph–COOH has a comparable value of BDE (429.7 kJ/mol) as Ph-CH_3_ [18], it has been shown that the presence of -OH facilitates the decarboxylation of hydroxybenzoic acid [19]. Since sodium salicylate has much lower stability than sodium benzoate, a similar destabilization by -ONa must be in play and the actual bonding strength of HOPh–COONa is expected to be low. Nevertheless, the subsequent facile decarboxylation might contribute more to the low thermal stability of sodium salicylate. 

The above analysis points the correlation between the thermal stability of molecules and the particular bond strength of Ar substituent. Thus, the thermal degradation might begin with the bond cleavage of Ar substituent. To test this assumption, TGA-FTIR of phenolates on the gaseous decomposition products and IR analyses on the residues were performed.

### 3.2. TGA-FTIR of Phenolates 

#### 3.2.1. Sodium Phenolate

**Figure 2:**
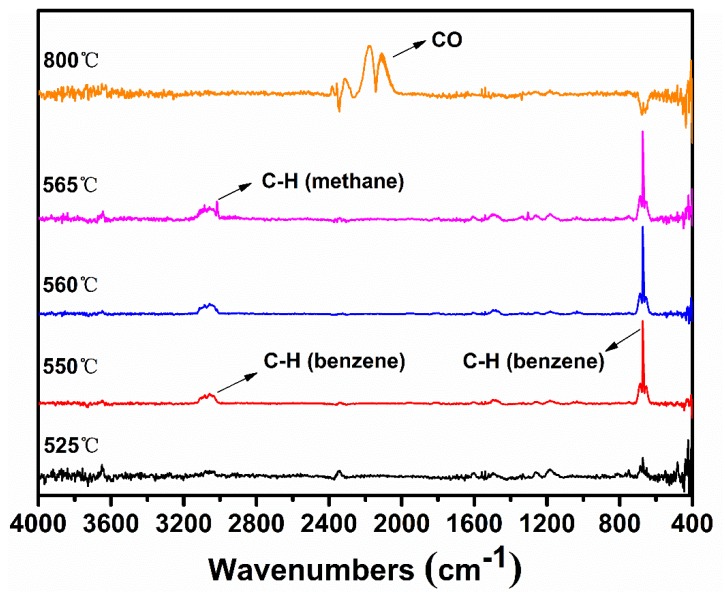
FTIR spectra of gaseous products of sodium phenolate at selected temperatures.

Sodium phenolate shows a surprisingly reduced thermal stability when compared with phenol which commences the degradation around 650 °C [20]. The negative charge of oxygen apparently accelerates or changes the degradation pathway of phenol. The thermal degradation of phenol is known to be initiated either by a homolytic cleavage of PhO–H or a tautomerization to keto forms [21,22]. In either case, cyclopentadiene and CO are major decomposition products. Since sodium phenolate does not possess -OH, the tautomerization to keto forms followed by the loss of CO and a simultaneous generation of cyclopentadienide would be envisaged. However, besides a small amount of phenol which is almost always present, the only detectable gaseous species is benzene during most of time in the main degradation step as seen in TGA-FTIR spectra shown in Figure 2. Benzene is also unequivocally identified by the MS spectrum as illustrated in Figure S1. CO becomes noticeable only after the end of the main degradation step. Thus, the change from -OH to -ONa must alter the initial degradation pathway of sodium phenolate. 

**Scheme 1:**
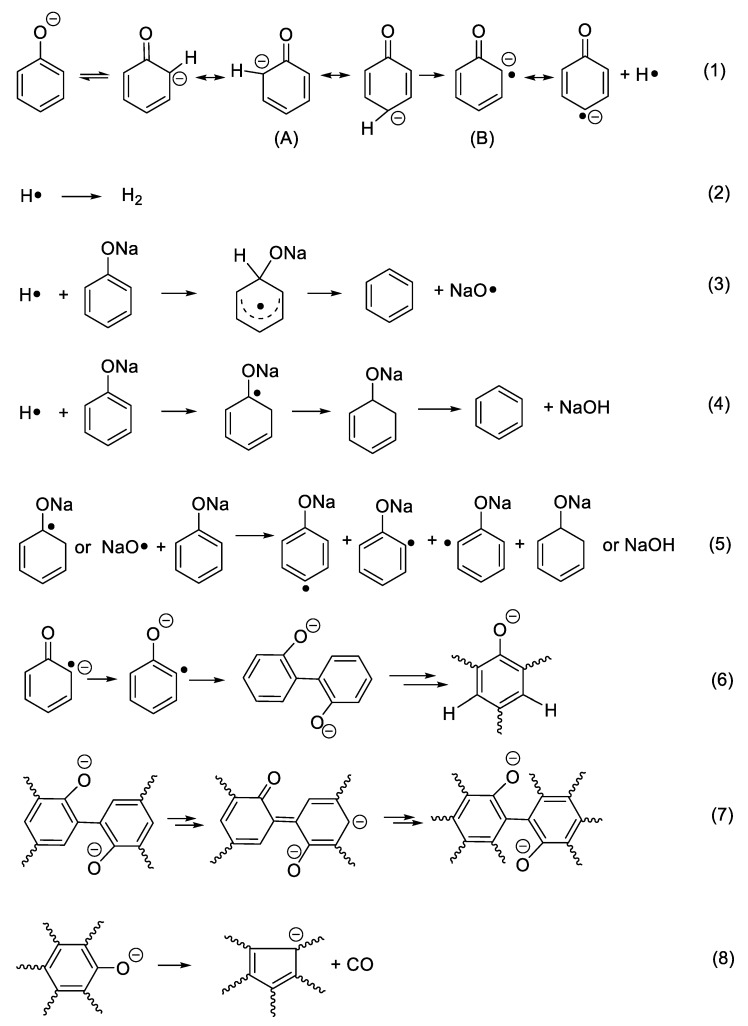
The aromatization process of sodium phenolate.

Considering benzene as the dominant species in the first stage, Ph–ONa must be broken. However, the homolysis of the bond is unlikely due to very high bond energy of Ph–O (459.0 kJ/mol for Ph–OH) [23]. Instead, an ipso attack of H· on C(-ONa) is assumed as it is proposed in the formation of benzene, a minor product, from the thermal degradation of phenol [24,25]. Nevertheless, in the case of phenol, H· can be conveniently produced from the homolytic cleavage of PhO–H. For sodium phenolate, aromatic H must be the source of H·. To account for this unexpected disconnect of Ar-H at a temperature much lower than that needed to break HOPh–H, it is hypothesized that the C(sp^2^)–H(*o*-, *p*-) bond is first weakened by the negative charge of oxygen and then cleaved to generate a carbon radical anion and H· as outlined in Scheme 1 (Reaction (1)). 

**Figure 3:**
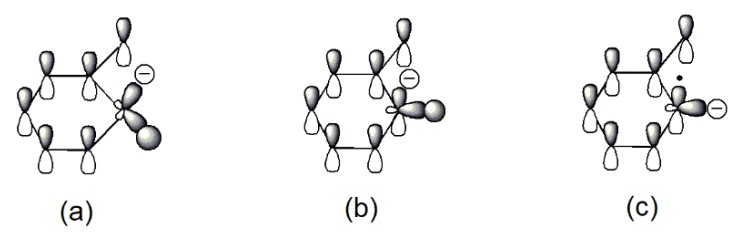
Illustrations of pi orbitals in keto form (A): (**a**) sp^3^ carbanion; (**b**) sp^2^ carbanion; and (B): (**c**) sp^2^ radical anion.

The relatively easy generation of the carbon radical anion (B) and its para- analogs in sodium phenolate can be rationalized as follows. Tautomerization of phenolate initially gives keto forms (A) that are an alkyl carbanion. Because the hybridization of an alkyl carbanion is normally sp^3^ and the geometry is pyramidal [26], the one sp^3^ orbital which is occupied by the negative charge is not in the maximum overlapping position with the adjacent pi orbitals due to the restriction of the space direction of orbitals and a reduced bond angle of (C–C^-^–C). Consequently, the negative charge of the alkyl carbanion largely localizes on the alkyl carbon as shown in Figure 3a. On the other hand, an allylic anion can adopt sp^2^ hybridization and delocalization of charge in the p orbital can take place with the neighboring unsaturated bonds [25]. However, for keto forms (A), although sp^2^ hybridization can give the p orbital where the negative charge locates a good overlap with the adjacent pi orbitals, the number of total electrons in the ring pi orbitals would be seven (Figure 3b). Thus, regardless of the type of hybridization of the carbanion, keto forms lose aromaticity. This is likely one of reasons that tautomerization from enol to keto forms is unfavorable. This unfavorable tautomerization is especially true for neutral phenol since there is a charge separation in the resonant keto forms [27], but should be less so in phenolate of which resonance structures only involve the movement of the negative charge.

To regain the aromaticity for keto forms (A), one way is to lose the hydrogen atom of the carbanion to transform to a radical anion (B). Since the hybridization of a carbon radical is sp^2^ [28], this leads to a planar geometry that allows a perfect overlap of the p orbital where the free radical is with the rest of ring pi orbitals as seen in Figure 3c. Furthermore, the number of total pi electrons in the ring is six now so an aromaticity is achieved in (B). Therefore, it is the resulted aromaticity of the radical anion that activates the NaOPh–H(*o*-, *p*-) bond. The above rationale about activation of NaOPh–H(*o*-, *p*-) is probably a bit simplified, but it is based on the solid commonly known knowledge and is easy to be used to analyze the thermal degradation of a variety of sodium phenolates as described below. 

After H· is generated, it can combine to H_2_ (Reaction (2)), of which presence is independently confirmed by the GC analysis of the collected off-gas (to see Figure S2), or it can add to the aromatic ring to displace -ONa and generate observed benzene and NaO· as shown in Reaction (3) (to see Figure S1). However, benzene could also be formed through a first hydrogenolysis of phenyl rings to cyclohexadiene radical followed by H abstraction and elimination of NaOH as shown in Reaction (4). The degradation of the hydrogenated structures eventually leads to the appearance of methane. On the other hand, NaO· or cyclohexadiene radical abstracts activated *o*-, *p*–Hs to produce aryl radicals (Reaction (5)). Combination of aryl radicals gives multi-substituted sodium phenolates (Reaction (6)), which can then activate *m*–H originally to -ONa according to Reaction (7). However, since multi-substituted sodium phenolates can be converted to multi-substituted benzene through Reactions (3) or (4), not all hydrogens are activated. In addition, some oxygenated aromatic hydrocarbons must survive the first degradation step since CO is detected at a high temperature as shown in Figure 2. CO can be generated via Reaction (8), following the similar degradation pathway of phenol at high temperatures. 

**Figure 4:**
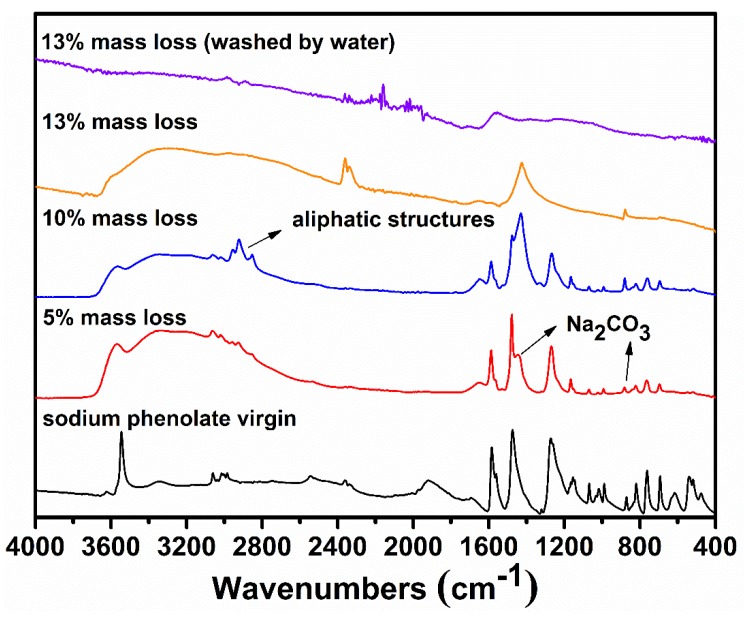
FTIR spectra of the residues of sodium phenolate at selected mass losses.

To gain further information on the degradation, the residues collected at selected mass losses were examined by FTIR spectroscopy and their spectra are shown in Figure 4. The most striking features at 5% and 10% mass losses are the appearance of strong absorptions at 2852, 2923, and 2955 cm^−1^, indicating the presence of C(sp^3^)–H. Since there is no aliphatic proton in sodium phenolate, the detections of aliphatic structures strongly support the hydrogenolysis of phenyl ring which can proceed as Reactions (4) and (5). The aliphatic features in the residue are clearly in line with the assumption that there exist active hydrogens and hence weak NaOPh–H bonds. In addition, a new peak growing with the mass loss appears at 1441 cm^−1^ and dominates in the spectrum of the residue collected after the main degradation step. This is found to be Na_2_CO_3_ which is apparently formed by the reaction of NaOH and CO_2_ in air. The spectrum of the water washed residue is nearly flat, suggesting the carbonaceous char in nature [29].

**Figure 5:**
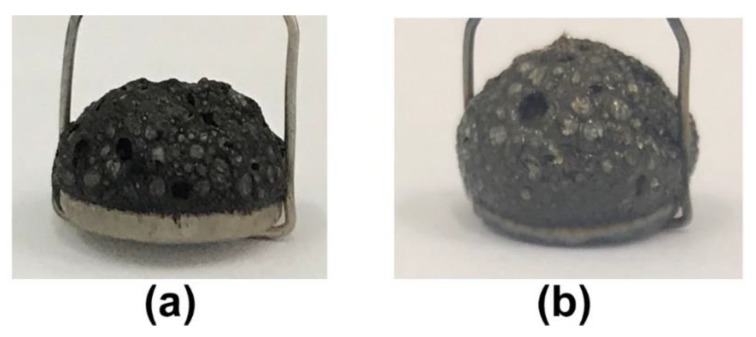
Intumescent char from (**a**): sodium phenolate; (**b**) sodium *p*-cresolate.

One highly interesting finding is that the residue collected after the main degradation step is intumescent as seen in Figure 5a. Traditionally, intumescence requires the presence of an acidic source [30]. The expanded carbonaceous char formation observed in sodium phenolate thus represents a completely different route to intumescence. Considering the mass loss of only about 13%, such an efficient charring process under a basic condition is highly remarkable and might be the key reason that alkali ions are good flame retardants for PC. In fact, intumescence has been noticed in PC/KSS flame-retardant system and considered to be a primary factor to contribute to the excellent flame retardancy of KSS [3]. Nevertheless, an acidic action of KSS has been suggested [31]. In view of the thermal degradation of PC that produces a large amount of phenols [32], it is reasonable to assume that the real action of KSS on PC stems from the intumescence of potassium phenolates which are generated from phenols and KSS or its decomposition products.

Compared to the thermal degradation of phenol, the removal of aromatic hydrogens in sodium phenolate is very fast. This is dramatically different from their extremely difficult loss in pure phenol even at a high temperature. In fact, the thermal degradation of phenol does not involve the bond cleavage of HOPh–H [33,34]. However, from the results of thermal degradation of sodium phenolate, it is safe to say that the degradation pattern of phenol can be fundamentally changed in the presence of alkalis. This change can be ascribed to the accelerated tautomerization to keto forms due to the strong electron-donating ability of oxygen anion. Furthermore, since the degradation of sodium phenolate generates sodium hydroxide, which can neutralize phenol and regenerate sodium phenolate, alkali can work as a highly efficient catalyst to change the decomposition pathway of phenols to one favoring an early aromatization. In this sense, a very small amount of alkalis will have a huge influence on the charring of oxygen-containing heavily aromatic polymers or biomass. This is the reason that only a trivial amount of KSS is required for PC to achieve an extraordinary flame-retardant rating. 

#### 3.2.2. Sodium p-Phenoxyphenolate

**Figure 6:**
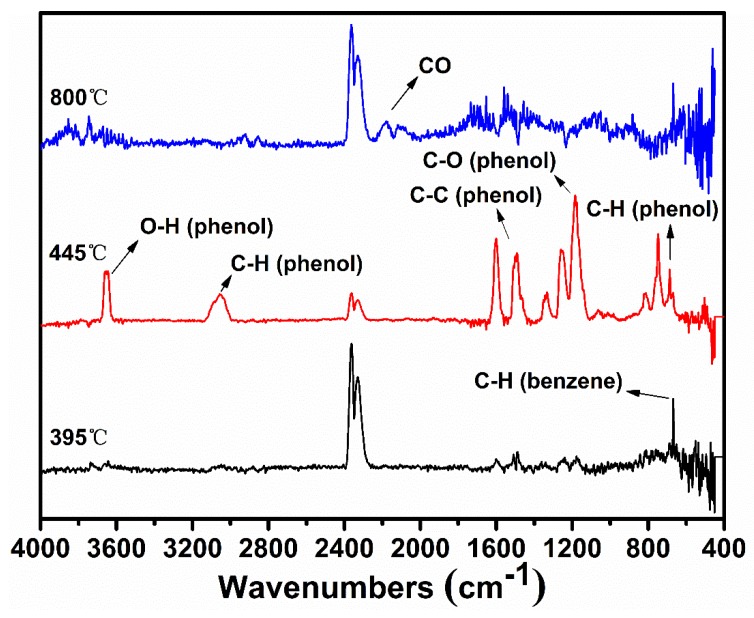
FTIR spectra of gaseous products of sodium *p-*phenoxyphenolate at selected temperatures.

Diphenyl ether is highly thermally stable up to 450 °C [35]. However, substitution of its one *p*–H by -ONa noticeably decreases the thermal stability of the molecule. The thermal degradation of *p*-phenoxyphenolate starts around 400 °C and loses 47.5% by weight in the main degradation stage as shown in Figure 1. Benzene and phenol are dominant gaseous products as seen in TGA-FTIR spectra in Figure 6. Phenol evolves in the entire first degradation step while benzene only shows up in the beginning of the decomposition. Theoretically, the mass loss due to phenol is 45.2%, so the first stage mainly involves the formation and vaporization of phenol. CO is merely found at the high temperatures after the main degradation step. 

**Scheme 2:**
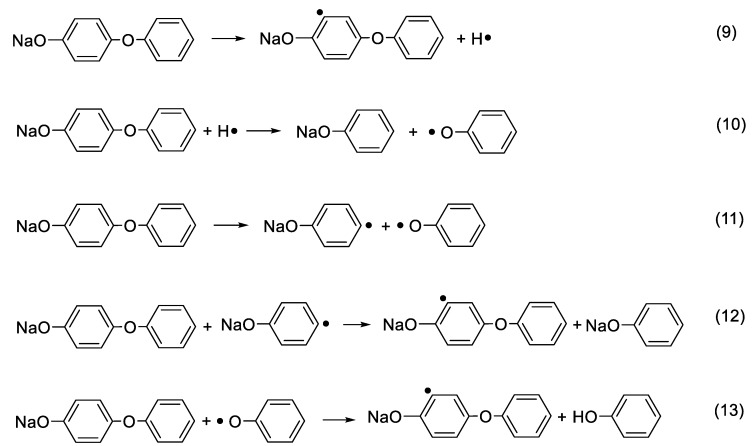
The aromatization process of sodium *p*-phenoxyphenolate.

In terms of the degradation mechanism, following the proposal in sodium phenolate, the initial decomposition of *p*-phenoxyphenol can begin with the homolysis of either NaOAr–H(*o*-) or NaOAr–OPh or both. If the initiation begins with the homolytic cleavage of NaOAr–H(*o*-), the resulted H· adds to a second phenoxyphenol molecule and displaces phenoxy group forming sodium phenolate and ·OPh as shown in Scheme 2 (Reactions (9) and (10)). On the other hand, the homolysis of NaOAr–OPh yields NaOAr· and ·OPh. NaOAr· abstracts hydrogens to yield NaOPh as illustrated in Reactions (11) and (12) or undergoes Reactions (6) and (7) to crosslinking structures. In both cases, sodium phenolate and ·OPh are common intermediates. While sodium phenolate is quickly responsible for the observation of benzene, the fate of ·OPh is very interesting.

It has been reported that PhO· degrades to yield cyclohexadiene radical and CO [36]. However, since CO is not observed in the main degradation step, it necessitates that the degradation of PhO· is very slow at 400–450 °C. On the other hand, the abstraction of labile hydrogens by PhO· readily accounts for the detection of phenol as shown in Reaction (13). In this sense, the bond strength of NaOAr–H (*o*-) can be estimated to be even weaker than PhO–H. Given a much higher BDE of the virgin Ph-H than that of PhO–H (361.9 kJ/mol) [17], the magnitude of this bond activation of NaOAr–H (*o*-) is enormous.

Compared with sodium phenolate, sodium *p*-phenoxyphenolate is much less thermally stable. This is in line with the weak bond of Ar–OPh which generates phenoxy radical either by direct homolysis or hydrogenolysis. On the other hand, sodium phenolate loses the first step mass by breaking down the relatively strong bond of NaO–Ph via hydrogenolysis. Thus, it is the easy generation of phenoxy radical that leads to an early mass loss of sodium *p*-phenoxyphenolate. 

#### 3.2.3. Sodium p-Cresolate

*p*-Cresolate has three weak bonds. Like sodium phenolate, Ar–CH_3_ and Ar–H (*o*-) are activated by -ONa. Besides these two bonds, ArCH_2_-H has a relatively low BDE (PhCH_2_–H = 368.2 kJ/mol). However, considering that NaOAr–H (*o*-) is weaker than PhO–H, the homolytic cleavage of ArCH_2_–H should not be competitive here. 

**Figure 7:**
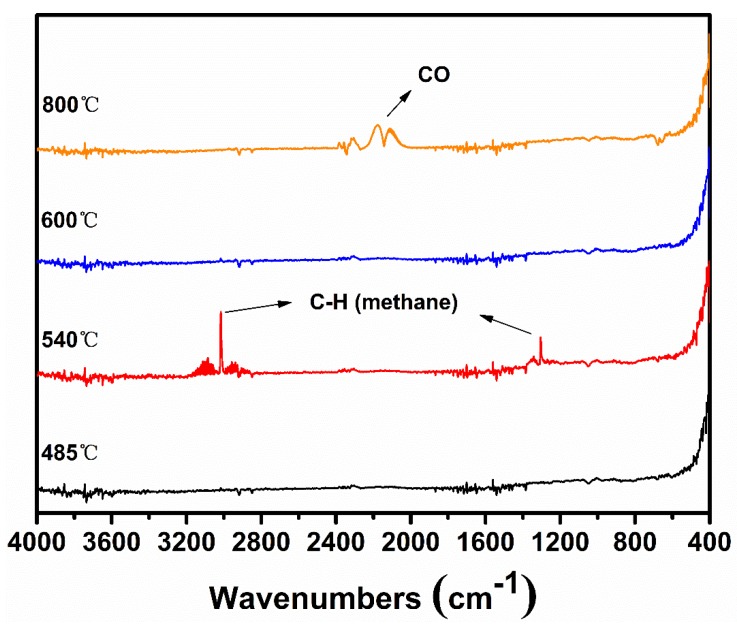
FTIR spectra of gaseous products of sodium *p-*cresolate at selected temperatures.

**Scheme 3:**
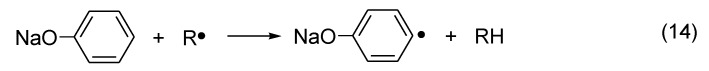
The dependence of lifetime of phenolate intermediate on the reactivity of abstracting radicals.

Figure 7 shows the TGA-FTIR spectra of off-gas from the thermal degradation of sodium *p*-cresolate. It can be clearly seen that methane is the only gaseous product in the main degradation step. The theoretical value of mass loss due to methane is 12.3%, close to the 13.2% obtained in the TGA curve. Thus, the first degradation step mainly involves the demethylation. The presence of methane can be promptly accounted for by the initial homolysis of Ar–CH_3_ or Ar–H (*o*-) or both, just like the generation of phenol from *p*-phenoxyphenolate. However, it is interesting to note that unlike the thermal degradation of *p*-phenoxyphenolate, benzene is not found in sodium *p*-cresolate. Since the formation of benzene requires a pre-generation of sodium phenolate, the absence of benzene suggests that the lifetime of phenolate is very short. Given the much higher activity of CH_3_· than PhO·, Reaction (14) in Scheme 3 would be faster when R· = CH_3_· so virtually little sodium phenolate is formed during the thermal degradation of *p*-cresolate.

On the aspects of residue, sodium *p*-cresolate also produces an intumescent char at the end of the main degradation step as seen in Figure 5b. In addition, the IR spectra are nearly identical to those from sodium phenolate as judged from Figure S3. Overall, sodium phenolate and sodium *p*-cresolate share a similar aromatization process. 

Finally, it is interesting to compare the demethylation of sodium *p*-cresolate with that of *p*-cresol. In the case of *p*-cresol, methane is formed only after the fragmentation of *p*-cresol to CO and methylcyclopentadiene. It is the further degradation of methylcyclopentadiene that generates methane [18]. On the other hand, CO is not observed before or together with methane during the main thermal decomposition step of sodium *p*-cresolate. Instead, methane is produced mainly by the first disconnect of NaOPh–CH_3_ followed by the hydrogen abstraction of CH_3_·. Thus, the routes to methane are profoundly different from *p*-CH_3_Ph–OH to *p*-CH_3_Ph–ONa.

#### 3.2.4. Sodium m-Cresolate

Sodium *m*-cresolate shows a similar degradation pattern as sodium *p*-cresolate. There does not seem to have an apparent influence of different positions of methyl group in the ring on the mass loss and type of degradation products. Methane is the only detected gaseous product in the main degradation step. However, the initial bond break in *m*-cresolate must start from the homolytic cleavage of Ar–H(*o*-, *p*-). After the generation of H·, it adds to a second molecule to form sodium phenolate and CH_3_·, which abstracts activated hydrogen to yield methane and aryl radicals. The subsequent degradation pathway is analogous to that of sodium *p*-cresolate. The IR spectra of the residue collected after the main degradation step are also virtually identical to those of the residue from *p*-cresolate (to see Figure S3). 

#### 3.2.5. Sodium Salicylate

**Scheme 4:**
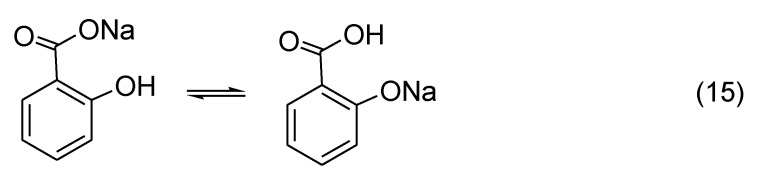
Equilibrium of the phenolate form and carboxylate form in sodium salicylate.

Sodium salicylate has a great propensity to undergo thermal degradation. It starts to decompose at ~240 °C, significantly lower than sodium benzoate that is stable to at least 425 °C as illustrated in Figure 1. The easy decomposition of sodium salicylate might come from the phenolate form which is in equilibrium with the carboxylate form as shown in Scheme 4 (Reaction (15)). 

**Figure 8:**
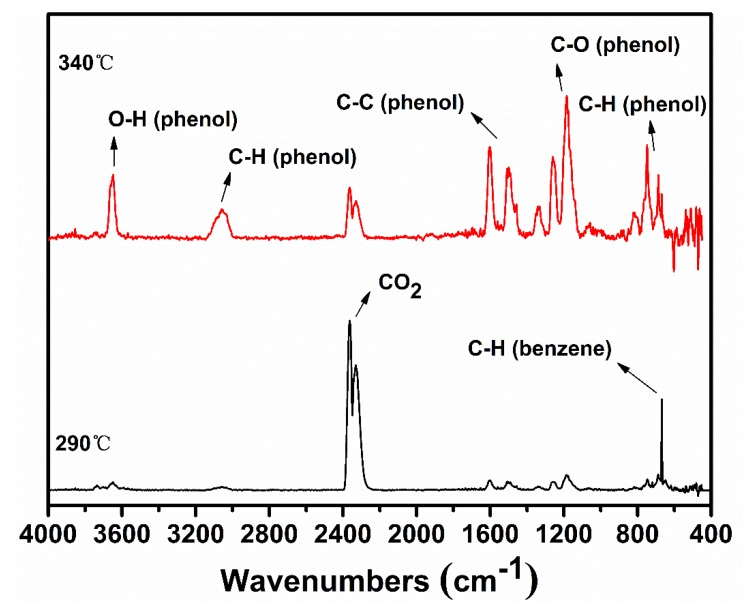
FTIR spectra of gaseous products of sodium salicylate at selected temperatures in the first decomposition step.

The phenolate form apparently activates Ar–H(*o*-, *p*-) and Ar–COOH bond of which homolytic cleavages yield aryl radicals and H· or ·COOH, respectively. The latter can foreseeably decompose to CO_2_ and H·. H· and aryl radical then undergo Reactions (2–7). Due to the formation of sodium phenolate and the absence of an active alkyl radical, generation of benzene is expected. H· can also add to sodium salicylate and produces phenol. The presence of both benzene and phenol is confirmed in the first decomposition step by the TGA-FTIR spectra shown in Figure 8. 

## 4. Conclusions

Thermal degradations of a series of sodium phenolates have been investigated. The replacement of -OH by -ONa considerably lowers the thermal stability of phenolates. The thermal stability of phenolates is generally in parallel with the bond strength of NaOPh–R. The weaker the bond is, the easier their decompositions are. The major gaseous degradation products in the main step are those derived from substituents or benzene. This degradation pattern is significantly different from that of phenol.

To account for the reduced thermal stability of sodium phenolates and the degradation products a mechanism involving an aromatic radical carbanion has been proposed. It is based on the notion that the localized or delocalized carbanion of keto forms need to homolytically lose a substituent to achieve an aromaticity. The homolytic cleavage of Ar substituent/H(*o*-, *p*-) is greatly enhanced by the strong electron-donating ability of oxygen anion, which rationalizes the difference in the thermal degradation pathways of phenol and phenolates.

The detections of benzene in the gaseous phase and aliphatic structures in the residue from the thermal degradation of sodium phenolate strongly support the existence of active hydrogens and hence the weak NaOPh–H bonds. Furthermore, the preferred formation of phenol instead of degradation to cyclopentadiene radical and CO from phenoxy radical generated in the thermal degradation of *p*-phenoxyphenolate suggests that the bond strength of NaOAr–H(*o*-, *p*-) is even weaker than that of PhO–H. 

Overall, the aromatization process of phenolates is initiated by the homolysis of NaOPh–R(/H)(*o*-, *p*-). Succeeding hydrogen abstractions and aryl radical combinations lead to the crosslinking and eventually the charring. 

Considering the importance of base catalyzed char formation in the flame retardancy of polymeric materials and the efficient use of biomass as feedstock, understanding the origin of the alkali effect is critical. Our work on the degradation mechanism of model compounds, i.e., sodium phenolates, clearly shows that this alkali effect originates from the activation of Ar–H bonds by oxygen anion and will pave a way to take advantage of this base effect.

## Figures and Tables

**Table 1:** TGA data of sodium salts.

Sodium salts	T_1%_ (°C)	T_5%_ (°C)	T_max_ (°C)	Mass Loss (%)
phenolate	375	487	527	13.2
*m-*cresolate	359	489	501	13.0
*p-*cresolate	344	478	500	13.2
*p-*phenoxyphenolate	253	392	400	47.0
salicylate	246	253	261	42.9
benzoate	457	477	516	46.9

## Supplementary Materials

The following are available online at https://www.mdpi.com/2073-4360/11/7/1119/s1. Supplementary materials associated with this article can be found in Figure S1–S3.
