# Supplementary material for: On the Origin of Alkali-Catalyzed Aromatization of Phenols
Source: Polymers (Basel). 2019 Jul 2;11(7):1119. doi: 10.3390/polym11071119 (PMC6680450; doi:10.3390/polym11071119)
Supplement: Supplementary file 1 [file polymers-11-01119-s001.pdf]

Supplementary Information

On the origin of alkali catalyzed aromatization of phenols

Yu Ji[1,2], Qiang Yao[1,2,*], Yueying Zhao[1], Weihong Cao[1]

[1]*Ningbo Institute of Materials Technology and Engineering, Chinese Academy of Sciences, Ningbo, Zhejiang 315201, China*

[2]*University of Chinese Academy of Sciences, Beijing 100049, China;*

[*]Correspondence: yaoqiang@nimte.ac.cn

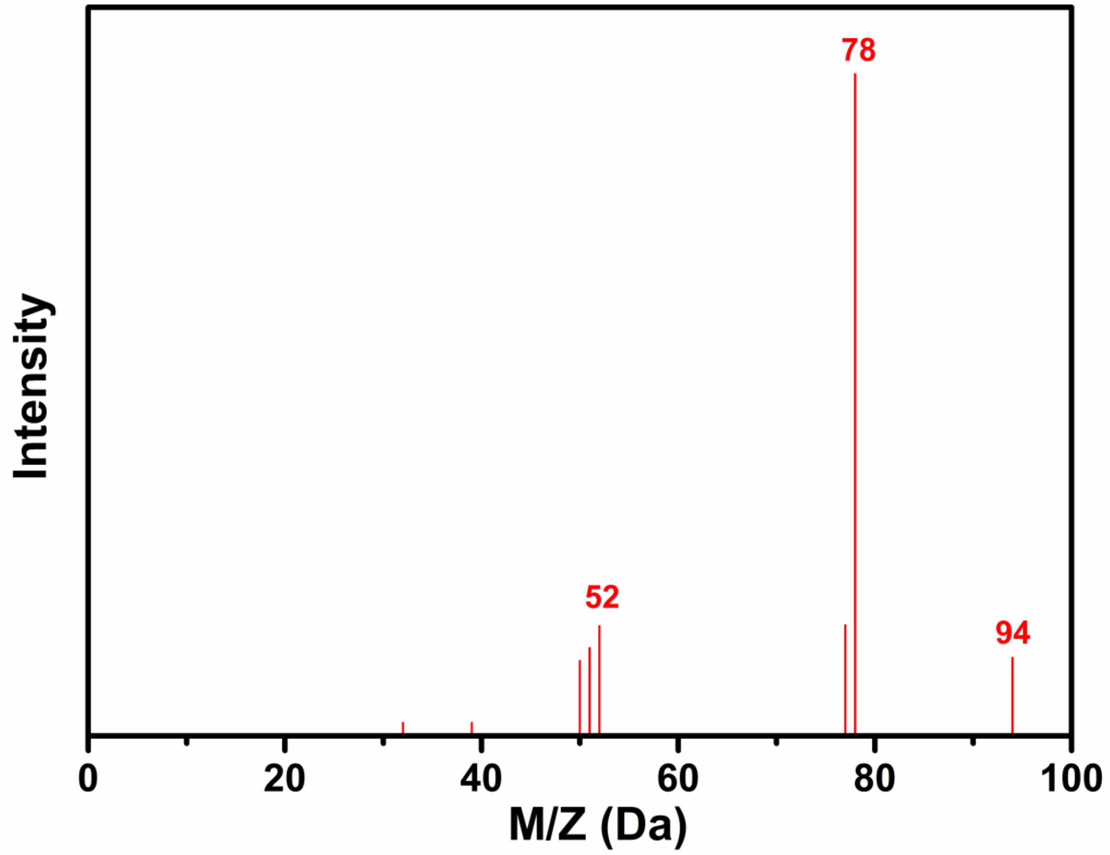

Figure S1. Mass spectrum of gaseous products of sodium phenolate at 550℃.

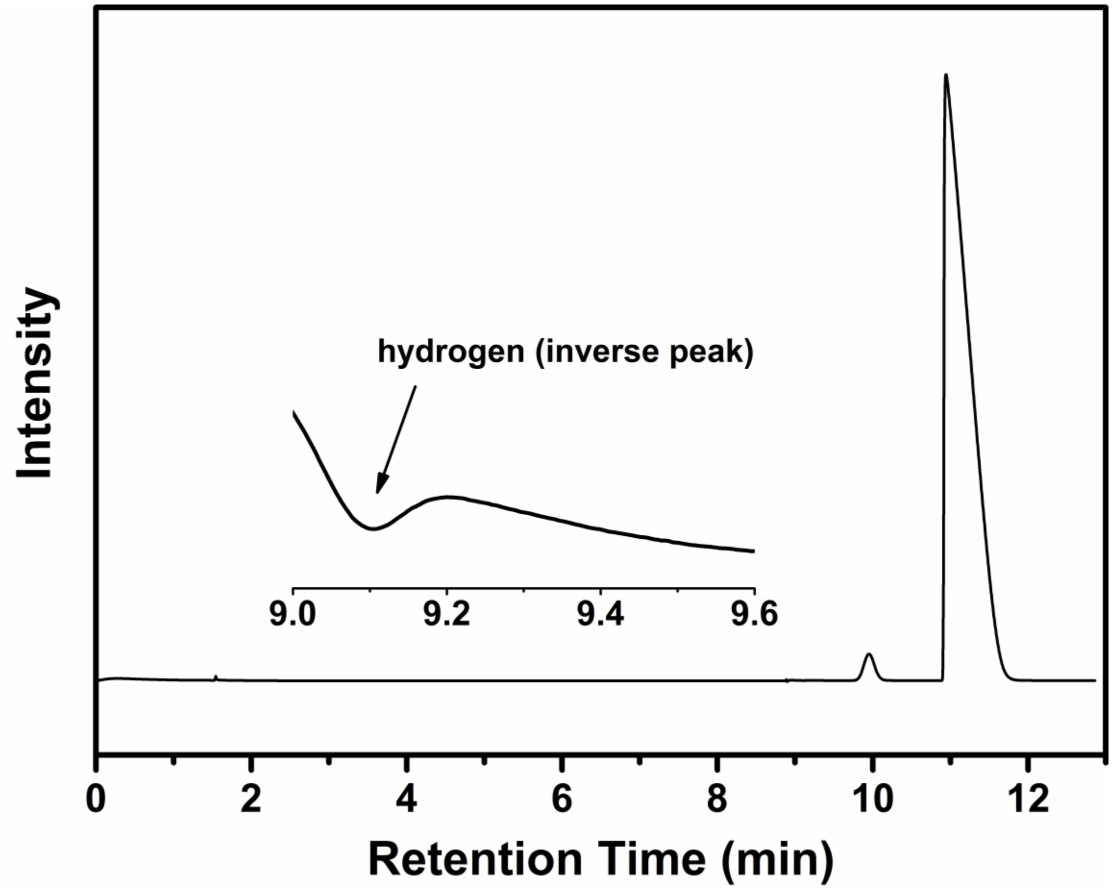

Figure S2. GC spectrum of the collected off-gas of sodium phenolate.

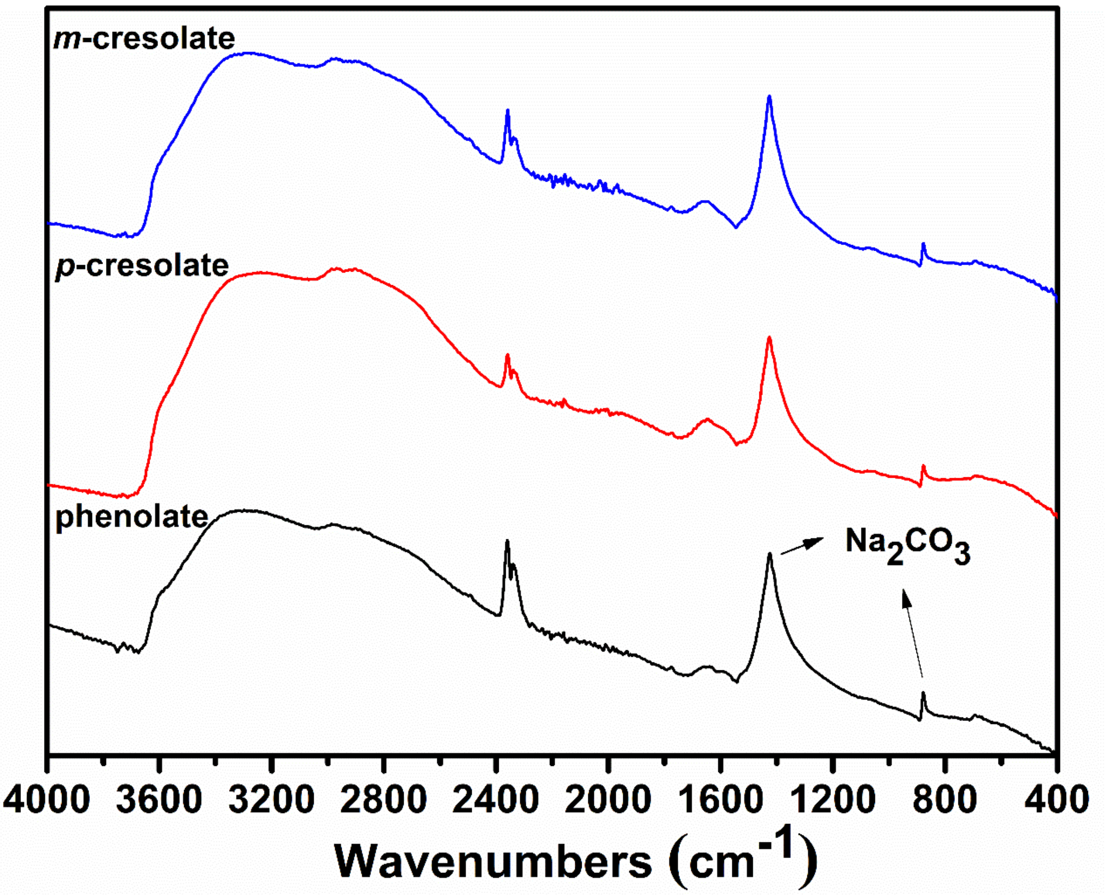

Figure S3. FTIR spectra of the residues collected after the main degradation steps of sodium phenolate, *p*-cresolate and *m*-cresolate.
